# A Case of Primary Aldosteronism Turned Out to Be Adrenocortical Carcinoma With Disorganized Steroidogenesis

**DOI:** 10.7759/cureus.52137

**Published:** 2024-01-11

**Authors:** Yohsuke Ohkubo, Yasuho Shimada, Hiroki Tanaka, Masanori Yamazaki, Mitsuhisa Komatsu

**Affiliations:** 1 Diabetes, Endocrinology, and Metabolism, Shinshu University, Matsumoto, JPN

**Keywords:** steroid enzyme immunostaining, cortisol hypersecretion, disorganized steroid production, adrenocortical carcinoma, primary aldosteronism

## Abstract

Adrenocortical carcinoma (ACC) is a rare disease with a poor prognosis, which essentially needs an early diagnosis because surgery is the only hope of a cure. On the other hand, primary aldosteronism (PA) is an overproduction of aldosterone from the adrenal glands and is known as one of the most common causes of secondary hypertension and hypokalemia. It is mostly a benign disease. ACC accompanied by PA is extremely rare, which can result in delayed diagnosis and clinical pitfalls.

A 56-year-old woman was diagnosed with PA. Mild, symptomatic PA was clinically diagnosed as a right-sided aldosterone-producing adenoma (APA) with adrenal tumor using adrenal vein sampling (AVS). The tumor imaging findings showed abnormalities on computed tomography (CT) in terms of size and attenuation value compared with typical benign adenomas. Twelve months later, the tumor was confirmed to be an ACC with cortisol hypersecretion. The resected ACC specimen did not clearly show positive findings for CYP11B1 or CYP11B2, and disorganized steroid production was suspected. However, the prevalence and clinical characteristics of adrenocortical carcinomas with disorganized steroid production remain unclear. Steroidogenic enzyme immunostaining analysis is important not only for the diagnosis of adrenal adenoma but also for a better understanding of the clinical course of hormone-producing ACC.

## Introduction

Steroid enzymes play a critical role in adrenal gland function. The precise categorization of primary aldosteronism (PA) is now achievable [1], with increasing emphasis on its clinical correlation. However, only a few cases of adrenocortical carcinoma (ACC) have been evaluated using steroid enzyme immunostaining. Herein, we present a case in which ACC with abnormal cortisol production developed after a diagnosis of PA, and hormone production was assessed using steroid enzyme immunostaining.

## Case presentation

**Figure 1:**
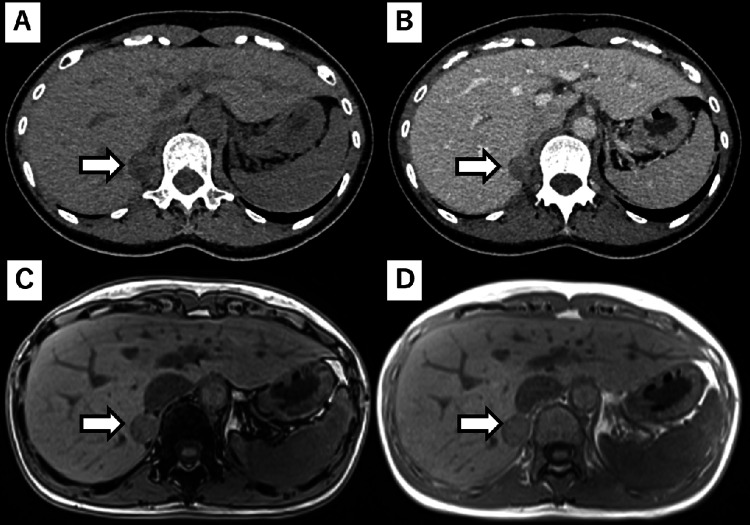
Adrenal gland tumor images at the PA diagnosis Right adrenal tumor images at the time of PA diagnosis were obtained using unenhanced CT (A), enhanced CT (B), out-of-phase MRI (C), and in-phase MRI (D). PA: Primary aldosteronism.

A previously healthy 50-year-old woman presented to the emergency department with a fever. A computed tomography (CT) study revealed a 20-mm right adrenal tumor (Figure 1, Panel A). The patient’s fever disappeared within a few days without treatment. The cause of the fever could not be identified. Thereafter, the patient visited an endocrinologist to examine the adrenal tumor. She had normal blood pressure (133/66 mmHg) and mild hypokalemia (3.5 mmol/L; reference range: 3.6-4.8). The patient had an elevated serum aldosterone level (267 pg/mL; reference range: 29.9-159) and suppressed serum renin activity level (<2.0 pg/mL; reference range: 2.5-21). Dehydroepiandrosterone sulfate (DHEA-S) levels were within the normal range (44 ng/mL; reference range: 8-188). A 1-mg dexamethasone-suppression test yielded a normal response (serum cortisol 0.65 µg/dL; reference range: <1.8). Other adrenal hormone levels were within normal limits.

A captopril challenge test subsequently confirmed the diagnosis of PA. Imaging studies showed a 20 × 20 mm right adrenal mass. The tumor had 32 HU on non-contrast CT, delayed washout on contrast-enhanced CT, and was lipid-rich on magnetic resonance imaging (MRI) (Figure 1). These findings, except for the CT attenuation value and size, and washout delay on contrast-enhanced CT were suggestive of an adenoma. Adrenal vein sampling (AVS) confirmed excessive aldosterone secretion from the right adrenal gland within the tumor before and after adrenocorticotropic hormone (ACTH) stimulation. Therefore, the patient was diagnosed with a right unilateral aldosterone-producing tumor.

**Figure 2:**
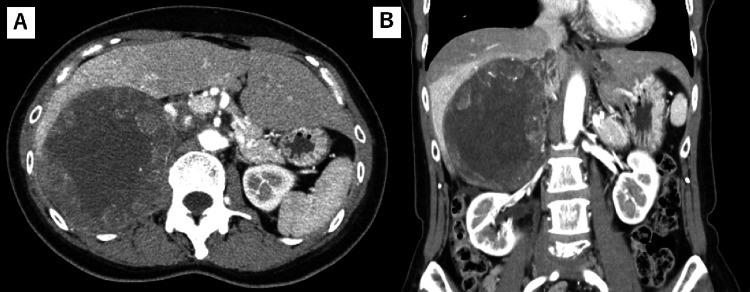
Adrenal gland tumor images before the adrenalectomy Right adrenal tumor images were obtained just before the adrenalectomy using enhanced CT in the transverse plane (A) and the coronal plane (B).

Based on the diagnosis and imaging findings, an adrenalectomy was required. However, surgery was delayed for personal reasons. The patient remained asymptomatic and was taking two antihypertensive drugs daily: 5 mg cilnidipine and 50 mg spironolactone. However, imaging performed 12 months later revealed that the tumor had grown to 130 mm in size (Figure 2). The patient had no additional symptoms such as anorexia, high blood pressure, and a Cushingoid appearance. Her blood tests just revealed elevated levels of lactate dehydrogenase and alkaline phosphatase as well as mild anemia due to iron deficiency. The test results of eosinophils, HbA1C, or lipids profile were normal. Serum aldosterone, renin activity, cortisol, and ACTH levels were 200 pg/mL (reference range: 35.7-254), 1.2 pg/mL (reference range: 3.2-36.3), 12.2 µg/dL (reference range: 1.0-15.0), and 4.7 ng/mL (reference range: 8-188), respectively. The urine-free cortisol level was 171 µg/day (reference range: 11.2-80.3). Furthermore, diurnal fluctuations in cortisol were absent, and a 1-mg dexamethasone-suppression test showed no suppression of cortisol secretion. Therefore, the patient was diagnosed with PA and ACTH-independent Cushing's syndrome.

Right adrenalectomy was performed because the tumor was considered to be an ACC. Histopathological examination revealed diffuse positivity for steroidogenic factor 1 (SF-1) within the tumor tissue, indicating an adrenal cortical origin. The Weiss score was positive in 7/9 points, and the Ki-67 labeling index was 36%. These findings confirmed the diagnosis of ACC. After the adrenalectomy, the plasma aldosterone and serum DHEA-S concentrations had decreased to 55.4 pg/mL (reference range: 35.7-254) and 11 pg/mL (reference range: 8-188), respectively, and the suppression of renin activity was reduced (7.1 pg/mL; reference range: 3.2-36.3). Steroid replacement therapy was initiated because of low cortisol levels.

**Figure 3:**
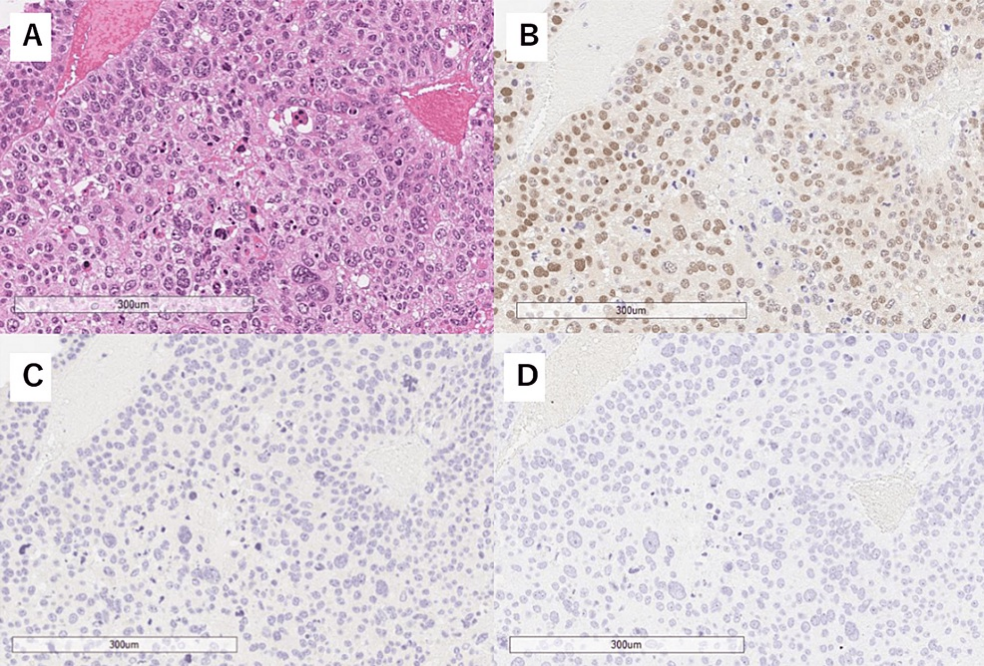
Pathology of the adrenocortical carcinoma Immunohistochemistry of the tumor cells. Hematoxylin & eosin (H&E) staining (A), steroidogenic factor 1 (SF-1) (B), CYP11B1 (C), and CYP11B2 (D), all at 100x magnification.

Immunohistochemical staining revealed that cholesterol side-chain cleavage enzyme (SCC), 3β-hydroxysteroid dehydrogenases (HSD3β), and dehydroepiandrosterone sulfotransferase (DHEA-ST) were expressed in some tumor cells, whereas both C21 and C17 were expressed in many tumor cells. CYP11B1 and CYP11B2 expression was rarely observed (Figure 3).

Mitotane treatment was initiated soon after adrenalectomy. However, the patient developed pulmonary metastases from the ACC. No severe hypertension or hypokalemia was present, although the metastatic tumors were growing. The patient experienced a decrease in both appetite and the activity of daily living after starting treatment with mitotane. Moreover, at the end of her life, the patient suffered from cachexia, anorexia, and chronic fatigue but no pain or severe hypertension. The patient was transferred to another hospital to receive hospice care and passed away 19 months after surgery.

## Discussion

Here, we present a case of ACC in which the tumor was clinically considered to be an aldosterone-producing adenoma (APA) with mild symptoms. Immunostaining raised the important question of where hormones such as aldosterone and cortisol are produced.

CYP11B1 and CYP11B2 are required for the production of cortisol and aldosterone, respectively. In the resected tissue of the current patient, these enzymes were not adequately detected pathologically. However, tumor removal resolved the excess hormone level problem. The resected tissue predominantly contained ACC cells, with only a minimal amount of surrounding tissue. Hence, we concluded that the ACC, rather than any other lesions, was responsible for cortisol production. We surmised that the discrepancy between immunostaining and the clinical course was caused by disorganized steroidogenesis.

Disorganized steroidogenesis is a steroid hormone production process unique to ACC that was first reported by Sasano et al. [2]. Unlike adrenal adenomas, ACC cells do not express all of the enzymes necessary for steroid production. Cells harboring distinct steroid enzymes are arranged in a disorganized manner. As a result, hormones are no longer efficiently produced in the ACC [2,3], which may lead to a mismatch between tumor size and hormone production capacity as well as alterations in ACC hormone secretion at metastatic sites. One previous report of disorganized steroidogenesis described a cortisol-producing ACC that was negative for CYP11β [2], similar to the present case. Moreover, in another patient, disorganized steroidogenesis was identified in a substantial ACC, measuring 7.2 cm in diameter, with mild PA and subclinical Cushing's syndrome [4]. The use of immunostaining in additional ACC cases may provide a better understanding of the disorganized steroidogenesis associated with ACC and its clinical characteristics.

The specific site of aldosterone production remains clinically and pathologically unclear. ACC or other undiscovered microadenomas can induce excessive aldosterone production. The characteristics of aldosterone-producing adrenocortical carcinomas (APAC), a rare disease, have only been evaluated in a limited number of patients. Seccia et al. reviewed 58 APAC cases and reported no obvious differences in tumor size, hormones, or symptoms [5]. ACC is typically suspected when the size reaches 4 cm or more [6]. Notably, 9% of APAC patients have presented with tumors measuring ≤ 3 cm [5]. Based on these findings, the possibility that the adrenal tumor was APAC at the time of the initial diagnosis could not be ruled out. Moreover, even after the diagnosis of ACC, we could not clinically exclude the possibility of aldosterone production from ACC because the lack of exacerbation of hormone overproduction by tumor growth or metastasis would not be helpful in the differential diagnosis. Normal plasma aldosterone and serum potassium levels were previously reported in two patients with APAC metastases [7,8]. Decreased hormone production capacity is a relatively common phenomenon after ACC metastasis, which may be attributed to hypodifferentiated alterations within the ACC [5] or metastasis of the non-producing cells associated with disorganized steroidogenesis. Furthermore, from a pathological perspective, disorganized steroidogenesis in APAC may prevent CYP11B2 positivity. Finally, considering the possibility of APA, the only way to completely rule them out is to pathologically prove their absence in substantial tissues. However, the tissue sample was too large for thorough examinations.

Determining whether the tumor was an ACC at the time of PA diagnosis is a pivotal question. Adrenal tumors, which are small in size and rich in fat, are usually treated as benign [6]. In the present patient, the tumor was different in size, CT attenuation value, and washout delay on contrast-enhanced CT compared to typical benign adenomas, typically characterized by dimensions smaller than 1 cm, CT values below 10 HU, and early washout [6]. In addition, high-fat concentration on MRI and normal range of DHEA-S were consistent with benign features [6]. We measured hormones from the adrenal gland preoperatively using blood samples to investigate changes in hormonal dynamics of the ACC; however, there was a limitation in obtaining meaningful results based on actual steroidogenesis in the tumor because of the influence of the steroidogenesis in the non-tumor tissue. Considering these observations, a definitive diagnosis of ACC at the time of PA diagnosis was challenging. Shortening the interval between follow-up imaging tests should be performed to address this issue if the tumor is not a typical adenoma.

## Conclusions

In this report, we presented a novel case of ACC complicated by disorganized steroidogenesis. The evaluation of pathological hormone secretion using immunostaining for steroid enzymes is important for explaining adrenal hormone secretion in clinical settings. We believe that future research investigating the clinical and pathological aspects of disorganized steroidogenesis will improve the prognosis of ACC.
